# Factors Associated with Adherence to Treatment with Isoniazid for the Prevention of Tuberculosis amongst People Living with HIV/AIDS: A Systematic Review of Qualitative Data

**DOI:** 10.1371/journal.pone.0087166

**Published:** 2014-02-03

**Authors:** Titilola Makanjuola, Henock B. Taddese, Andrew Booth

**Affiliations:** 1 Society for Family Health, Abuja, Nigeria; 2 School of Health and Related Research (ScHARR), University of Sheffield, Sheffield, United Kingdom; Institut de Pharmacologie et de Biologie Structurale, France

## Abstract

**Objective:**

To systematically identify from qualitative data in the published literature the main barriers to adherence to isoniazid preventive therapy (IPT) for tuberculosis (TB) among people living with HIV/AIDS (PLWHA).

**Methods:**

We searched ten data sources, including MEDLINE and EMBASE for articles published in peer-reviewed journals from inception through to December 2011 for evidence relevant to IPT for TB in relation to PLWHA. Studies were assessed for quality using the CASP critical appraisal tool for qualitative studies. Data extracted from studies were then analysed thematically using thematic synthesis.

**Results:**

Eight studies, two of which were conducted within the same clinical trial, met the inclusion criteria. In addition to the influence of personal characteristics, five overarching themes were identified: Individual personal beliefs; HIV treatment and related issues; Socio-economic factors; Family and other social support factors, and Relationships with health providers. The review confirms current understanding of adherence to treatment as influenced by patients' understanding of, and beliefs related to treatment regimens. This is in-turn influenced by broader factors, namely: socio-economic factors such as poverty and lack of health facilities; the level of support available to patients from family and other networks and the stigma that emanates from these relationships; and relationships with health providers, which in-turn become a delicate issue given the sensitivity of dealing with two chronic diseases of significant morbidity and mortality toll. HIV treatment related issues also influence adherence to IPT, whereby challenges related to the acceptance, organisation and administration of these two long-term treatment regimens and stigma related to HIV/AIDS, are seen to be major factors.

**Conclusion:**

Understanding this complex interplay of factors more clearly is essential for healthcare decision-makers to be able to achieve the level of adherence required to effectively mitigate the threat posed by co-infection with TB and HIV/AIDS in developing countries.

## Introduction

Tuberculosis (TB) is the most common opportunistic infection and leading cause of mortality in people living with HIV/AIDS (PLWHA). In PLWHA, the risk of developing TB is 21–34 times greater than those without HIV infection [1]. Globally, around 1.1 million people were estimated to be co-infected with HIV and TB in 2010, representing in excess of 10% of the 9 million new cases of TB that year [1]. This overall trend differs according to the state of the HIV epidemic in different settings. In hard hit areas such as Sub-Saharan Africa (where there is a generalized HIV epidemic), PLWHA represent around 39% of new TB cases [1]. Co-infection with HIV and TB resulted in some 0.35 million TB attributable deaths amongst people living with HIV worldwide, in the year 2010 [1].

The interaction between HIV and TB is bidirectional with each disease potentiating the adverse effects of the other. This, in turn, affects the prognosis of patients and complicates clinical diagnosis and treatment plans through atypical presentation of symptoms, adverse drug reactions, overlapping drug toxicities and drug-drug interactions between Highly Active Anti-Retroviral Therapy (HAART) and anti-TB drugs [2], [3], [4]. Co-infection with HIV and TB adds significantly to the burden on health systems in the developing world and complicates and threatens efforts aimed at achieving globally set development and health objectives [2], [3], [4], [5].

Isoniazid preventive therapy (IPT) for people living with HIV, who do not have active TB, is one of the strategies recommended by the World Health Organization (WHO) and the Joint United Nations Programme on HIV/AIDS (UNAIDS) to enable the effective prevention, diagnosis and treatment of TB in PLWHA [6], [7], [8]. The recommended regimen for TB preventive therapy in adolescents and adults is isoniazid (isonocotic acid hydrazide – INH), 300 mg daily for at least 6 months [8], [9]. A Cochrane review assessing the effectiveness of TB preventive therapy in reducing the risk of active TB and death in persons infected with HIV, ‘confirms that chemoprophylaxis with anti-tuberculosis drugs reduces the risk of clinical tuberculosis in HIV infected populations’ [10]. The review also cautions about the dangers of ‘poor adherence and drug resistant TB disease potentially associated with the use of long courses of isoniazid monotherapy’ [10].

Various health system related constraints have impeded the uptake of IPT. Only 12% of PLWHA who were newly enrolled in HIV care programmes were started on IPT, worldwide, in 2010 [1], [9]. Adherence to treatment is a critical factor that needs to be considered in scaling up services in developing countries [9], [10], [11]. This paper seeks to systematically review the evidence on adherence to IPT with a view to assessing and interpreting deterrent and enabling factors associated with observed trends in non-adherence.

## Methods

### Search strategy

The P-population I-Intervention C-Comparator O-Outcome framework is commonly used for determining inclusion and exclusion criteria for systematic reviews of quantitative studies [12]. For this review of qualitative data, a counterpart labelled as the ‘SPICE framework’ [13] was modified to formulate the review question. SPICE stands for S-setting, P-perspective, I-intervention, C-comparator and E-evaluation and specifies the key attributes of the review question (Table 1).

**Table 1:** SPICE formulation for the question.

**S**etting(s): Hospital or clinics administering IPT
**P**erspective: People living with HIV/AIDS (PLWHA)
**I**ntervention: Isoniazid preventive treatment
**C**omparison: None
**E**valuation: Factors that contribute to IPT adherence

We developed a highly sensitive search strategy combining key terms that may indicate the use of isoniazid (e.g. isoniazid/e), combined with the concept of adherence (e.g. adheren* OR complian*) and with an indicator of data required (e.g. qualitative OR findings OR interview*). This broad search strategy for qualitative data has been shown to perform acceptably when compared with more exhaustive lists of qualitative terms [14], [15]. Because the search was intended to be as sensitive as possible we did not restrict by population at the searching stage. Instead we established an explicit association of relevant papers with both HIV/AIDS and TB when sifting by title and abstract. Initial searches were developed (T.M.) for the following sources from inception to August 2011: Ovid MEDLINE, Embase, PubMed, CINAHL via Ebsco, Cochrane Central Register of Controlled Trials (Central), Scopus, Proquest, Web of Knowledge, Google and Google Scholar. Subsequently, a qualified and experienced information professional (A.B.) constructed a series of supplementary search strategies and methods to validate the initial retrieval set and to extend data coverage until December 2011. This process, which involved subject searching of MEDLINE and Web of Science and citation searches for included studies on Web of Science and Google Scholar, identified two additional recent studies. The search strategy for MEDLINE on OvidSP is provided as supporting information: Ovid MEDLINE Search strategy, Table S1. Our search was complemented by reviewing reference lists of relevant papers.

### Study selection

One investigator (T.H.) performed a preliminary scan of titles and abstracts for eligibility according to predefined inclusion criteria. Final decisions, for confirmation or in cases of uncertainty, were resolved in discussion with a second investigator (H.B.T). Title and abstracts from supplementary searches, identified by A.B. were examined by both the investigators (T.H. and H.B.T.) and an authoritative decision on inclusion was made jointly according to the original inclusion criteria. Once all potentially relevant full-text articles and abstracts were identified, we consulted as a team (T.H., H.B.T., A.B.) to achieve consensus regarding final eligibility.

### Data extraction

Initial data extraction was conducted independently, using a standardized form (T.H) and then verified by a second reviewer (A.B.). Subsequent tabulation was verified by a third investigator (H.B.T.). Data abstractors collected information about the study setting, study populations, sample size, dosage, and any mechanisms for ensuring adherence. Our primary interest was in verbatim responses from informants on barriers to their adherence with isoniazid preventive therapy. However we were also interested in patient responses to pre-identified factors, for example in structured surveys or questionnaires. For triangulation purposes only we also decided to include reports from health personnel when in direct contact with patients and who, therefore, had perceptions on reasons for adherence/non-adherence. However these were treated as “indirect evidence” only and, therefore, were not used to direct our initial conceptual model. We applied the Critical Appraisal Skills Programme (CASP) checklist for qualitative studies, as adapted by Hawker and colleagues, since it can also be used in studies where disparate data are involved [16]. The nine detailed questions were answered according to the responses; Good, Fair, Poor and Very Poor to arrive at an overall qualitative judgement on study quality.

### Data synthesis

Several alternative methods exist for synthesis of qualitative data. Where studies contain conceptually-rich data and the objective is to improve theoretical understanding, more interpretative methods, such as meta-ethnography, are available. However, the output of some methods of synthesis, (e.g. thematic synthesis and framework synthesis), is considered more directly relevant to policymakers and designers of interventions than outputs from methods with a more constructivist orientation (e.g. meta-ethnography) which are generally more “complex and conceptual” [17]. Thematic synthesis is analogous to methods of primary analysis of qualitative data such as thematic analysis, using techniques to formalize the identification and development of themes [18]. Such themes can be explored both individually and in terms of their inter-relationships with each other. Thematic synthesis was therefore used to analyse factors relating to adherence with isoniazid preventive therapy in people living with HIV/AIDS.

## Results

**Figure 1:**
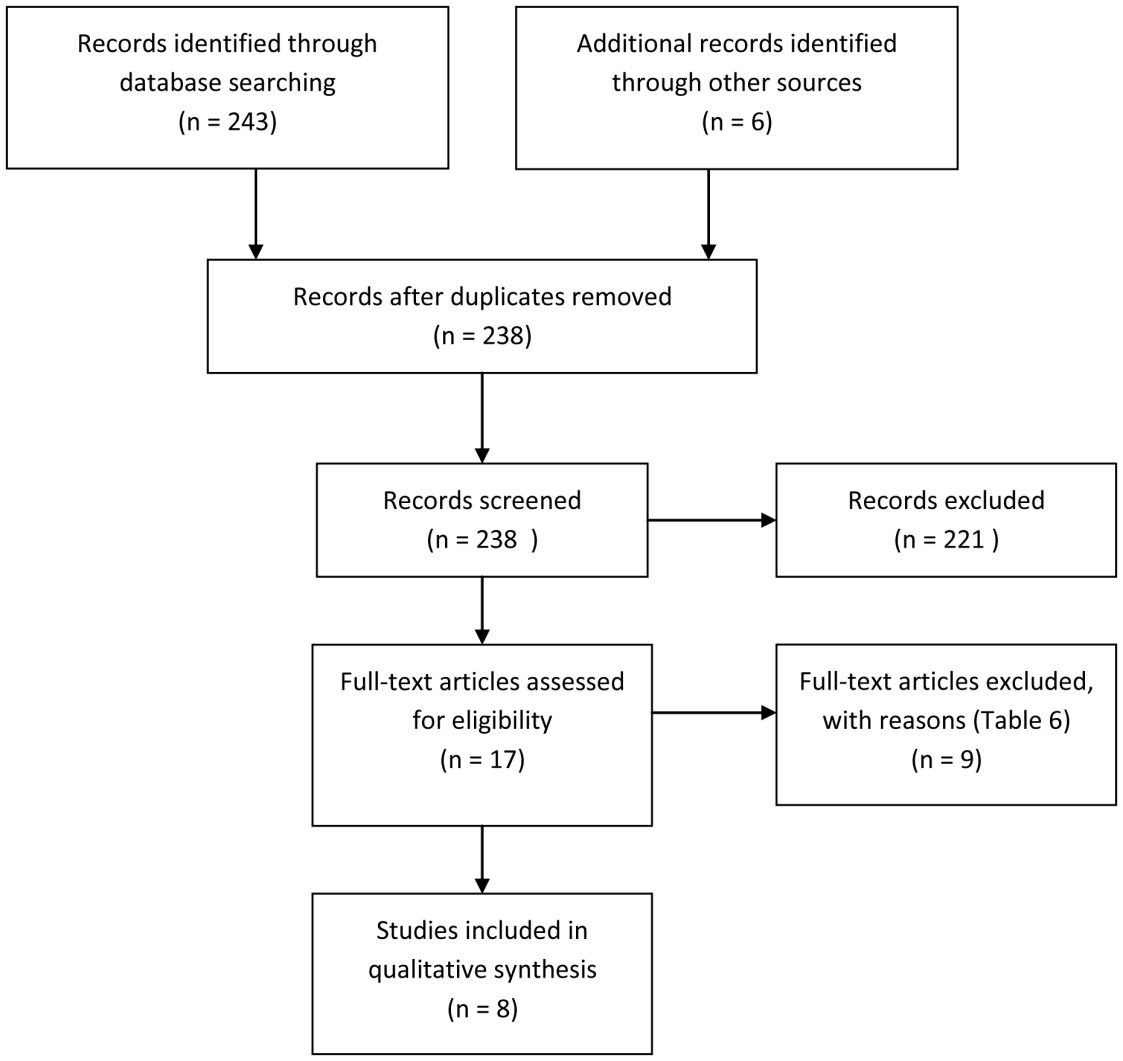
PRISMA flow diagram of selection of studies.

Figure 1 shows the flow diagram of study selection for analysis. Furthermore, the nine studies excluded at the final stage of the selection process, after assessment of the full text articles, are presented in table 2, along with the reasons for exclusion.

**Table 2:** Excluded studies with reasons for exclusion.

Excluded studies	Reason(s) for Exclusion
Grant AD, Charalambous S, Fielding KL, Day JH, Corbett EL, Chaisson RE, De Cock KM, Hayes RJ, Churchyard GJ. Effect of routine isoniazid preventive therapy on tuberculosis incidence among HIV-infected men in South Africa: a novel randomized incremental recruitment study. JAMA. 2005 Jun 8;293(22):2719–25.	No adherence related data
le Roux SM, Cotton MF, Golub JE, le Roux DM, Workman L, Zar HJ. Adherence to isoniazid prophylaxis among HIV-infected children: a randomized controlled trial comparing two dosing schedules. BMC Med. 2009; 7:67.	Not Adult perspective
Okanurak K, Kitayaporn D, Akarasewi P. Factors contributing to treatment success among tuberculosis patients: a prospective cohort study in Bangkok. Int J Tuberc Lung Dis. 2008;12(10):1160–5.	Adherence to IPT was not assessed
Hiransuthikul N, Nelson KE, Hiransuthikul P, Vorayingyong A, Paewplot R. INH preventive therapy among adult HIV-infected patients in Thailand. Int J Tuberc Lung Dis. 2005 Mar;9(3):270–5.	Only tests significance of Tuberculin Skin Testing (TST) as a factor in adherence
Lertmaharit S, Kamol-Ratankul P, Sawert H, Jittimanee S, Wangmanee S. Factors associated with compliance among tuberculosis patients in Thailand. J Med Assoc Thai. 2005;88 Suppl 4:S149–56.	Adherence to IPT was not assessed
Lester R, Hamilton R, Charalambous S, Dwadwa T, Chandler C, Churchyard GJ, Grant AD. Barriers to implementation of isoniazid preventive therapy in HIV clinics: a qualitative study. AIDS. 2010;24 Suppl 5:S45–8.	Only studies barriers to implementation
Nuwaha F. Factors influencing completion of treatment among tuberculosis patients in Mbarara District, Uganda. East Afr Med J. 1997;74(11):690–3	Reported elsewhere
O'Brien RJ, Perriëns JH. Preventive therapy for tuberculosis in HIV infection: the promise and the reality. AIDS. 1995;9(7):665–73.	Case report
Souza CT, Hökerberg YH, Pacheco SJ, Rolla VC, Passos SR. Effectiveness and safety of isoniazid chemoprophylaxis for HIV-1 infected patients from Rio de Janeiro. Mem Inst Oswaldo Cruz. 2009;104(3):462–7.	No analysis of factors for non-adherence

### Settings and Populations

The eight included papers [19], [20], [21], [22], [23], [24], [25], [26] were published between 1997 and 2011 and were conducted in Botswana [20], [22], Ethiopia [21], South Africa [25], [26], Tanzania [19], [23], and Thailand [24]. Close examination revealed that two of the studies were based on data from the same clinical trial that sought to ‘determine whether continuous isoniazid is superior to a 6 month course…’ [20], [22]. The earlier published paper is a secondary analysis of trial records and its objectives related to adherence were to find correlations between observed adherence rates on the one hand, and patient characteristics and influences of ART on the other [22]. The latter published paper involved administration of surveys and focus group discussions and in-depth interviews amongst a sample of adherents and non-adherents [20]. Data from both papers were therefore extracted for this synthesis. Details of data extracted from all studies are presented in tables 3 and 4.

**Table 3:** Study characteristics of included studies.

Study information	Ngamvithayapong (1997) [24]	Bakari (2000) [19]	Rowe (2005) [25]	Szakacs (2006) [26]	Munseri (2008) [23]	Mosimaneotsile (2010) [22]	Gust (2011) [20]	Mindachew (2011) [21]
**Country**	Thailand	Tanzania.	South Africa	South Africa	Tanzania	Botswana	Botswana	Ethiopia
**Setting**	HIV provincial clinic, Chiang Rai	Different police stations in Dar es Salaam,	Bohlabela District Hospital	2 HIV clinics in Pietermaritzburg	Muhimbili University of Health	8 public health clinics in 2 largest cities in Botswana	8 public health clinics in Botswana	4 hospitals in Addis Ababa
**No. of Participants**	412 Persons Living with HIV	400HIV-1 infected police officers	87 HIV-positive clinic attendees	301 consecutive HIV positive patients	565 HIV-infected subjects in DarDar TB vaccine trial	1995 PLWH recruited to trial and attending government clinics	462 PLWH recruited to trial and attending government clinics	319 HIV positive individuals attending TB/HIV clinics of selected hospitals
**Study Design**	Qualitative and Quantitative	Quantitative nested study from prospective follow up cohort	Qualitative and Quantitative	Quantitative	Qualitative and Quantitative	Quantitative – clinical trial with follow up for adherence	Qualitative and Quantitative	Quantitative cross sectional study

**Table 4:** Intervention characteristics of included studies.

Study information	Ngamvithayapong (1997) [25]	Bakari (2000) [20]	Rowe (2005) [26]	Szakacs (2006) [27]	Munseri (2008) [24]	Mosimaneotsile (2010) [23]	Gust (2011) [21]	Mindachew (2011) [22]
**Intervention (Dose/Duration)**	Isoniazid (300 mg once daily and one tablet of vitamin B complex/nine months)	Isoniazid (300 mg once daily/six months)	Isoniazid (300 mg daily distributed on a monthly basis/six months	Isoniazid (300 mg daily) with pyridoxine	Isoniazid ((300 mg) and pyridoxine (50 mg) daily/six months)	Isoniazid (300 mg once daily/six months)	Daily tablets of isoniazid or placebo for three years and one tablet of vitamin B6.	Isoniazide at dose of 5 mg/kg, maximum dose of 300 mg daily/six months plus pyridoxine at fixed 25 mg daily dose.
**Adherence monitoring strategy**	Participants instructed to bring leftover medicine with them so that programme staff could count the pills.	Compliance assessed by pill counting	Not specified	Questionnaire and urine test strips for detection of INH metabolites	Not specified	Pills in returned bottles counted at months 1, 3, 6 and nurses used chart to assess adherence.	Study nurses provided bottles of medication and interviewed participants monthly, provided reminder cards and performed pill counts with participants on a quarterly basis.	Percentage adherence calculated by dividing number of pills taken by number of pills prescribed. Then, percentage adherence estimated by average adherence rate to drug
**Definition of adherence**	Proportion of those taking more than 80% of pills,	Adherence rate defined as taking more than 80% of the pills.	Completion of a full 6-month course.	Patients deemed to be adherent or non-adherent based on result of urine test	Non-completers separated into ‘physician-initiated’ (IPT discontinued by study physician because of side effects or new contraindications), ‘patient-initiated’ (those stopping medication on their own or did not return for follow up) and ‘died’.	Attending 6 monthly visits. Nonadherence defined as missing > = 1 visit during 6-month course of IPT or, for those who suffered death, adverse events or TB, missing >17% (one-sixth) of visits up until time of the event.	Non-adherence defined as refusing tablet ingestion but agreeing to quarterly physical examinations.	Patients reporting intake of 80% or more of prescribed medication over past 3 and 7 days considered adherent. Patient reporting intake of less than 80% of prescribed doses over past 3 and 7 days considered non adherent
**Other Notes:**			Most patients lived in villages and townships surrounding the hospital, although few within walking distance.	Hospital A - tertiary care in affluent suburban area; attendees pay US $3 for a consultation or medication. Hospital B - district hospital in impoverished suburban area, limited access to specialist care; significantly less modern facilities. Patients may attend either hospital's HIV clinic; same physicians staff both sites.	Subjects provided with travel reimbursement to be seen monthly thereafter for provision of medications and assessment of adherence and side effects.			

The eight studies employed qualitative and quantitative methods as well as mixed method designs. This synthesis focuses primarily on the qualitative data. PLWHA were recruited from in- and outpatient hospitals and clinics providing TB preventive treatment [20], [21], [22], [25], [26], from a cohort of PLWHA participating in a TB vaccine trial [23], HIV screening services for different population groups such as blood donors, counselling service users and those attending the routine provider initiated HIV Counselling and Testing Service (HCT) [24], as well as from cohort studies focusing on specific population groups such as commercial sex workers [24] and police officers [19]. Participants were screened with Tuberculin Skin Testing (TST) for TB infection before enrolment and were excluded if they had a history of TB or if they were hypersensitive to TST. Sputum examination for acid fast bacilli (AFB) and chest radiograph were performed in each case except for one study [20], where insufficient details were provided. Informed consent was obtained for each participant. One study involved commercial sex workers and blood donors [24], another involved police officers [19] while the others involved general populations of PWLHA.

A total of 4,228 patients were involved in the studies ranging from 87 to 1995 patients, with two studies [22], [23] having in excess of 500 patients. The basic demographic characteristics of study subjects were reported variably in the included studies. The sex distribution of study subjects was reported across the studies, whereby 2,796 (66%) of the total study population were females. The mean age was reported in (or could be retrieved from) the studies by Bakari et al [19], Gust et al [20], Mindachew et al [21], and Szakacs et al [26] as 38, 35, 35 and 33 years respectively. On the other hand, Mosimaneotsile et al [22] and Munseri et al [23] reported the median age in their study as 32 years. Ngamvithayapong et al [24] and Rowe et al [25] reported proportions of the study population within specified age groups whereby those above 35 years of age accounted for contrasting proportions of 15 and 77 percents, respectively.

For the studies that reported the marital status of study subjects [19], [21], [22], [24], 44% of respondents were married at the time of the survey. For studies that reported the employment status of study subjects [20], [21], [22], [23], the pooled proportion for unemployed subjects is found to be around 30%. While Ngamvithayapong et al report the employment status of their study population; they basically just report the proportion of those who are self employed (46%) and those employed by others (54%). As for the studies that reported educational status, the proportions of those who had a secondary level education or more were reported at more than 55% by three studies [20], [21], [22] while one study reported this at just 22% [24]. Other basic demographic characteristics reported in the studies include: monthly income, in variable currencies, by Mindachew et al in USD [21] and Gust et al in Botswana Pula [20]; Body Mass Index, only by Gust et al [20]; and distance to clinic, using variable measures of, ‘time to clinic’ by Gust et al [20] and subjective reports of how far respondents felt the clinics were, by Ngamvithayapong et al [24]. As such, the demographic characteristics were not reported in a standardised fashion across the studies and are only summarised here to add to the contextual understanding of the synthesis presented in this review.

### Study characteristics

Studies rated as better quality gave an in-depth account of the qualitative design used and the subsequent method of analysis [20], [25]. The apparently relatively poor quality of some studies was directly attributable to the poorer quality of their reporting [23], [24]. All studies performed well for applicability, with the exception of one study which was limited to a police officer population [19]. The utility of this particular study was further limited in only including analysis of factors for acceptance of enrolment in IPT and not factors for adherence or non adherence after commencement of the medication. Quality assessment of qualitative research studies is currently a contested area, with even poor quality studies potentially offering useful insights [18], so a decision was made not to exclude any studies on the basis of study quality alone. Table 5 shows the assessment of the methodological quality of included studies.

**Table 5:** Quality assessment of included studies.

Author Id Checklist Item	Ngamvithayapong (1997) [25]	Bakari (2000) [20]	Rowe (2005) [26]	Szakacs (2006) [27]	Munseri (2008) [24]	Mosimaneotsile (2010) [23]	Gust (2011) [21]	Mindachew (2011) [22]
Abstract and Title	Good	Good	Good	Good	Good	Good	Fair	Good
Introduction and aims	Good	Good	Good	Good	Fair	Fair	Good	Good
Methods and data	Good	Good	Good	Good	Good	Good	Good	Good
Sampling	Fair	Fair	Fair	Fair	Good	Fair	Good	Good
Data Analysis	Fair	Good	Good	Good	Good	Good	Good	Good
Ethics and bias	Fair	Fair	Fair	Fair	Good	Good	Good	Good
Findings	Good	Fair	Good	Good	Fair	Fair	Good	Poor
Transferability/Generalizability	Good	Poor	Good	Good	Good	Good	Good	Good
Implications and usefulness	Good	Poor	Good	Good	Good	Good	Good	Good

### Intervention

IPT was given to all participants orally for 6 months except in one study [24] where the duration was 9 months. Most studies stated that participants were prescribed with 300 mg INH except for one study [20] which did not state the amount given. Typically, participants were required to collect their drug supply on a monthly basis and were instructed to bring any left-over pills for assessment.

### Outcomes

Different strategies were used to assess adherence. Adherence was defined as self reported adherence over the last 7 days (reported adherence, 86.5%) [21], adherence to at least 80% of scheduled clinic visits for medicine refill (reported adherence, 78%) [20], completion of entire 6 months regimen of IPT in two studies [22], [23] (reported adherence, 87%, for both studies), urine tests for isoniazid metabolites (reported adherence, 72%) [26], taking more than 80% of pills (reported adherence, 65.5%) [19], patterns of pill collection (reported adherence, 47.1%) [25], and based on the 216 pill count cut off point (reported adherence, 67.5%) [24]. Accordingly, adherence rates ranged between 47.1% and 87%.

### Themes

Thematic synthesis involves identification of similar themes, organization of these similar themes into related areas and the development of analytical themes [17]. Major themes are constructed by reflecting, reading and re-reading, comparing, and interpreting the primary identified themes. Such major themes include verbatim data from respondents and interpretations put forward by the authors of included studies. Following critical analysis, all primary themes were categorized into sub-themes based on the review findings. Findings from this qualitative systematic review can be organised under five different overarching themes; (i) individual personal beliefs; (ii) HIV treatment and related issues; (iii) socio-economic factors; (iv) psychosocial and family factors, and (v) relationships with health providers (Table 6). Of these themes, only theme (ii), that is, HIV treatment and related issues, was specific to this particular population, when compared to a general population with TB [27]. Some issues, such as stigma, may be compounded where a patient has HIV in addition to TB, but conceptually the array of issues is similar. ‘HIV treatment and related issues’ is therefore prioritised within the following description of the themes, in order to highlight the unique contribution of this review.

**Table 6:** Major and sub-themes identified from included studies.

Theme/Subtheme	Sample Data
**1. Individual personal beliefs**	
a. Fear of INH side effects,	Perceived side effects of isoniazid [25]Side effects of the study medication (but personal doctor did not tell me to stop) [21, p. 7]I always felt like vomiting and my eyes were always itching because of the pills.” [21, p. 4]
b. Perceptions of HIV	People have noticed that everywhere they go, it says ‘HIV kills’. So even if I take treatment, I am not going to be cured. I am going to die …so that's why people cannot take treatment regularly’ [26, p. 266]
c. Belief in INH safety	22% agreed that INH is dangerous to your health [27, p. 5]Those who believed that INH was safe were less likely to have a negative urine test [27, p. 3]
d. Fear of TB/HIV complications	The 109 interviewed completers cited the following factors in their decision to complete IPT: fear of TB (n = 48, 44%)… fear of TB and HIV complications (n = 24, 22%) [24, p. 1039]
e. Knowledge of IPT importance	Misunderstanding about duration of the preventive therapy [25]Despite having completed the 9 month programme, about a quarter of the participants still did not know about the effect of isoniazid in preventing clinical TB [25]
f. IPT understanding	“I have completed the 9-month IPT programme and I do not know the effect of Isoniazid in preventing clinical TB, I think Isoniazid is dangerous to my health” [27, p. 4]
g. Being asymptomatic	‘Since last year I took the tablets for TB. Then I find I feel better, and I don't take the tablets. And even this year I took another package for TB. But when I feel better, I don't drink the tablets. Only when I feel pain.’ [26, p. 266]Health worker: ‘Really a person can't take medicine when he's not sick’ [26, p. 266]
h. Forgetting	Forgetfulness [22]Patients who reported they sometimes forget to take the INH were more likely to have negative tests [27,p. 4]
**2. HIV treatment and related issues**	
a. Denial of HIV status	Denial of HIV status [25]
b. HIV disclosure	‘It's not good to tell anyone…because it is spread all over the village’ [26, p. 265]
c. Concurrent use of HAART	“I was taking a lot of tablets and I was always thinking I will die…so I decided to stop these ones (isoniazid).” [21, p. 4]
d. Alternative treatments	‘They think that if they go to the traditional healers, they will give them something to drink. They are given a medicine, they think they will be cured.’ [26, p. 266]
e. Pill Burden	Taking too many pills [21, p. 7]
**3. Socio-economic factors**	
a. Out-migration for employment and competing work priorities	Out-migration for job search in other provinces [25, p. 110]“My job contract came to an end and I had to relocate to my home village” [21,p. 4]“The reasons were work commitments. My job was a barrier to taking the pill but the medication treated me well.” [21, p. 4]Many noted competing needs and priorities at home in relation to subsistence issues for themselves and their families. [26, p. 265]
b. Economic resource limitations	‘It's the money that can make people come and take tablets. Because of the distance, because people cannot just walk to the clinic, because you have to come every month … So if people can have money, there will be no problem.’ [26, p. 265]The limited resources available to this rural population were universally cited as a barrier to adherence [26, p. 265]Patients cited the need to use public taxis and the associated financial costs as a significant obstacle. [26, p. 265]
c. Economic dependence on family	People who depended on their parents or husbands for financial support, namely adolescents and women, remarked that the decision to come to the clinic was not entirely their own. One young woman stated: ‘It is difficult sometimes when my parents say they don't have money to give me to come to the clinic, for transport’ (new patient). [26, p. 265]When asked why she had difficulty coming to the clinic, another interrupter answered: ‘It was one day when I didn't come to the clinic because I didn't have the money to come.’ [26, p. 265]Young woman: ‘It is difficult sometimes when my parents say they don't have money to give me to come to the clinic, for transport’ [26, p. 265]
Distance from home to clinic	“We need to have a number of dispersed clinics so that people who are residing in rural areas get the medical services they need at the right times.” [21, p. 5]“When you are far from the clinic, the transport to the clinic becomes a problem.’ [21]Because of the distance, because people cannot just walk to the clinic, because you have to come every month…[26, p. 265]
Location of drug supply	“The fact that the clinic is private and separate from the general outpatient clinic, I can explain everything that is confidential and secret to me. It's a good place’ (new patient)”. [26, p. 266]
**4. Family and other social support related factors**	
a. Stigma/Double stigma	It's not good to tell anyone … because it's spread all over the village. So I'll be having a problem because I won't be free when I go around. I'll be afraid of the people’ (interrupter) [26, p. 265]“They (Botswana) still discriminate against people on the trial and that discrimination is what makes people drop out of the trial.” [21,p. 4]
b. Concern about family	Some said that they were concerned about their children and family, and these concerns motivated them to prolong their life including the taking of isoniazid. [25, p. 111]
c. Instrumental support from another family member	“I have an aunt in (a neighboring township). She is a businesswoman. She's the one who is taking care of me, she's helping me and she is very supportive of me even to come to the clinic, to join the support group, she's the one who motivated me to come here…. She gives me nutritious food” [26, p. 266]‘So those who stop, it's because they don't have someone who is taking care or controlling their treatment’ [26, p. 265]
d. Support group	‘I feel relieved just because I find myself with other people who are sick. We have this illness together.’ [26, p. 266]
e. Family responsibilities	Many noted competing needs and priorities at home in relation to subsistence issues for themselves and their families [26, p. 265]
f. Other social factors	Members of the church are taught that they cannot combine the clinic medication with (church) tea: She's got days for tablets and days for tea, not at the same time.’ [26, p. 266]
**5. Relationships with health providers**	
a. Clinic Environment	The fact that the clinic is private and separate from the general outpatient clinic helped to reduce patients' fear of stigmatisation: ‘But now, I can just explain everything that is confidential and secret to me. It's a good place’ [26, p. 266]‘…They [completers] were satisfied with the service and the providers’ [25]
b. Service availability	Drug supply: An alarming level of peripheral pharmacies are reported to run out of medications, which may impair overall adherence [27, p. 5]
c. Health provider relationship	The importance of supportive nurses for adherence was mentioned by almost all the completers, and their sentiments are summarized in the words of one woman who said: ‘… So again when I visit the clinic, I feel nice when I visit the clinic. Because when I get here they motivate me, encourage me not to think about it (HIV status) and the nurse tells me everything that I must not worry about. When I come back from seeing that nurse, I feel nice and I always want to visit that nurse because she's always telling me good things.’ [26, p. 266]
d. Physician advice	Personal doctor told me to stop because of medical problems including side effects of the study medication [21, p. 7]“Every time I did not understand, I asked and they made sure they explained clearly in order for me to understand better.” [21, p. 4]

### Theme one: Individual personal beliefs

Five studies [20], [23], [24], [25], [26] described individual personal beliefs as core factors contributing to the adherence of IPT. Such individual personal “micro-level” beliefs include fear of INH side effects, understanding of IPT and its importance, and belief in INH safety. Three studies [24], [25], [26] focused on patients' understanding of the effect and the repercussions of defaulting or not adhering to IPT treatment. Other related reasons include misunderstanding about the duration of IPT which also highlights the importance of counselling prior to enrolment [24]. However Ngamvithavapong and colleagues also observe that patients were successful in adhering even though they lacked accurate knowledge [24]. This is shown in the following statement:

*‘Some of them [adherents] thought that Isoniazid reduced blood HIV concentration or that it prevents other AIDS-related complications’* [24]

### Theme two: HIV treatment and related issues

HIV treatment and related issues such as concurrent use of HAART and denial of HIV status posed further contributing factors towards non-adherence to IPT. Patients in included studies reported feeling discouraged to take two regimens at the same time. They also described how they experienced toxicities, felt their disease was too advanced to be cured or did not want to be associated with the HIV “stigma” [20]. Denial or non-disclosure of HIV status was the most common of all sub-themes identified from the selected studies. This was a major theme in two studies [20], [25], as fear of rejection and stigmatization prevented people from disclosing their HIV status or coming out for treatment. This is confirmed both by author interpretation and by direct quotations from participants:

The reason for this difference may be that, in our setting, IPT is linked to HIV, and women in this study did not want their HIV status to be disclosed due to fear of separation from their spouses or families. [23]

*‘It's not good to tell anyone … because it's spread all over the village. So I'll be having a problem because I won't be free when I go around. I'll be afraid of the people’ (interrupter)* [25]

Beyond the influence of HIV related stigma, Rowe and colleagues draw upon a host of observations related to people's understanding and interpretation of HIV/AIDS as an illness, what they call, ‘an individual's health culture’ [25]. This relates to the perception of HIV/AIDS as incurable and the associated tension between people's faith in western medicine on the one hand and traditional healing practices on the other, which especially come to a head in the case of such ‘fatal’ diseases [25].

*“Members of the church are taught that they cannot combine the clinic medication with the (church tea)”* [25]

Switching to herbal medicine was also amongst reasons for non adherence discussed by participants in the study by Ngamvithayapong and colleagues [24]

### Theme three: Socio-economic factors

Socioeconomic factors were frequently associated with barriers to adherence. One aspect to this is the competition from other social and economic responsibilities. Participants in included studies describe the difficulties of maintaining a regular drug supply when required to participate in harvesting, military service or physically distant employment. Participants were more likely not to appear for treatment in cases where they had to seek permission from their employers. One participant suggested:

*“Even the bosses should be told about this program so that tomorrow when people ask for permission for these visits every month, they should know what is going on.”* [20]

Gust et al also state that, *‘themes associated with barriers to trial participation included, for example, competing commitments…and relocation’*. [20]

Four studies [20], [23], [25], [26] highlighted that patients experienced problems in accessing TB treatment because of distance, location of hospital or clinic, and non-availability of service providers. Conversely, participants in one study [25] gave instances of where the clinic environment positively influenced adherence because of its seclusion and privacy from other public places:

*“The fact that the clinic is private and separate from the general outpatient clinic, I can explain everything that is confidential and secret to me. It's a good place’ (new patient)”* [25]

### Theme four: Family and other social support related factors

Family and other social support related factors mainly include the nature of relationships with family members, the wider community, and others taking IPT, as well as the adverse effects of stigma that emanates from these relationships [25]. Relationships with family and friends appear to determine whether patients feel comfortable about taking IPT [20]. Stigma may also make patients ashamed to ask their employers for permission to attend their TB treatment. This impedes early treatment and facilitates progression of Latent to Active TB [24]. In some families, responsibilities of parenthood could work as a motivating factor to adhere to treatment while others reported that responsibilities such as taking care of children could reduce the possibility of adherence [26]. Support and encouragement from spouse, family and community health workers and concern for family members facilitated adherence to IPT as exemplified by the following data extracts from included studies:

Some said that they were concerned about their children and family, and these concerns motivated them to prolong their life including the taking of Isoniazid [24]

‘I feel relieved just because I find myself with other people who are sick. We have this illness together’ [25]

### Theme five: Relationships with health providers

The reception received by participants when attending health services [25], including whether effective communication takes place [20], has a major impact on the patient's adherence to treatment. Several extracts relate to the relationship with health providers, specifically in terms of the nature of the advice, and whether it's given or not given [21]. Rowe and colleagues reported that individuals most likely to benefit from HIV-related clinical interventions were those who were already users of health services [25]. They concluded that substantial challenges remain amongst those reluctant to present for testing, care, and support. In some cases, recruitment in a study may result in conflicting advice between the study personnel, who advocate compliance, and a personal physician who expresses reservations about the treatment [20]. Clearly such a tension juxtaposes a new and contingent relationship against a longstanding, perhaps even lifelong relationship with a personal doctor. Such a situation may be exacerbated where belief in the effectiveness of treatment is limited and patients are encouraged, either by family members or by others in the community, to explore more traditional alternative treatments [25].

### Framework for explaining factors influencing IPT adherence

**Figure 2:**
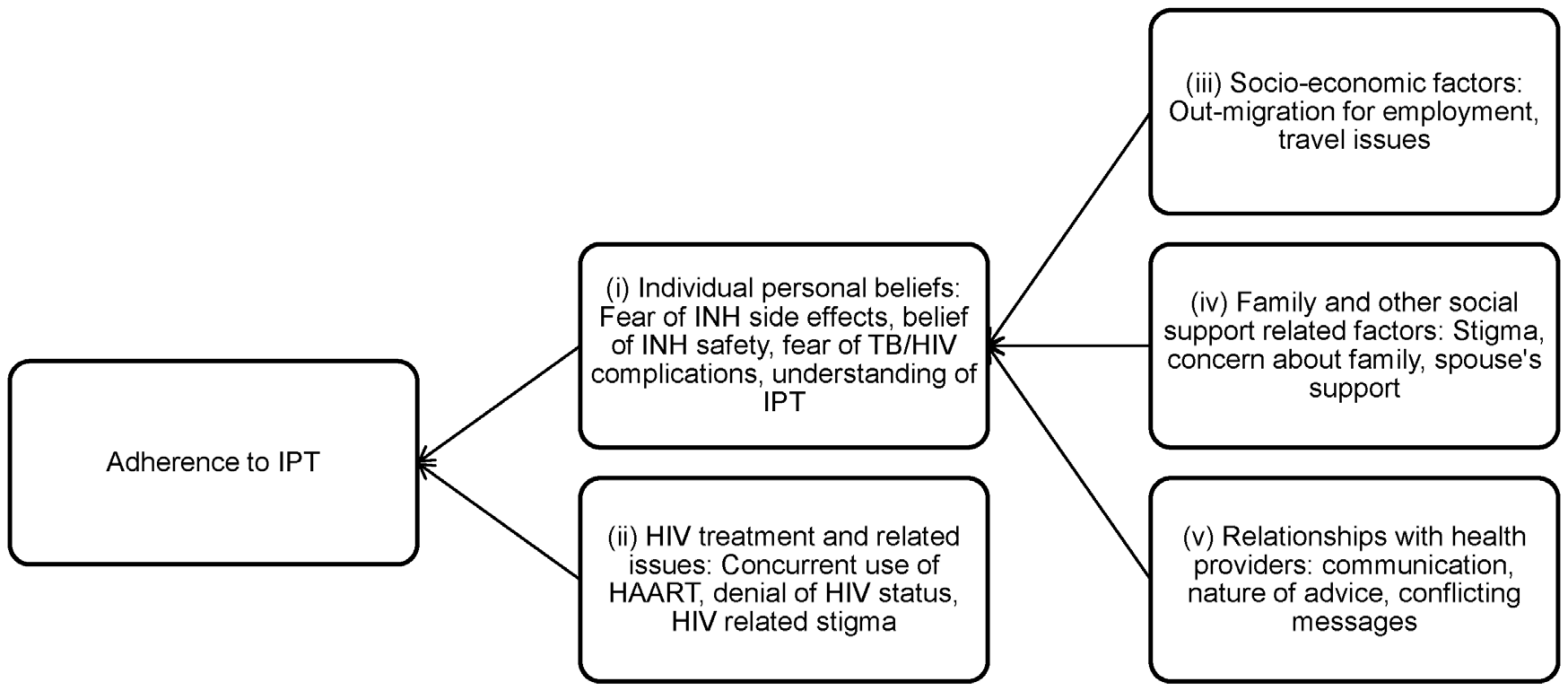
Conceptual framework of factors affecting adherence to IPT amongst PLWHA. A key interaction occurs between individual personal beliefs and family and social support factors; where patients' interactions with family and the wider community, including health workers (relationships with health providers), influence their knowledge, attitudes and beliefs about IPT treatment. Socio-economic factors are likely to influence individual personal beliefs, especially where patients live far from clinics and have fewer opportunities to be enlightened about the benefits of IPT. Similarly, HIV treatment and related issues affect patients' response to IPT treatment; where patients are not likely to adhere to IPT treatment if they are not willing or able to disclose their HIV status.

A model adapted and modified from Munro and colleagues represents how factors interact to influence IPT adherence [27]. A key interaction occurs between individual personal beliefs and family and social support factors; where patients' interactions with family and the wider community, including health workers (relationships with health providers), influence their knowledge, attitudes and beliefs about IPT treatment. Socio economic factors are likely to influence individual personal beliefs, especially where patients live far from clinics and have fewer opportunities to be enlightened about the benefits of IPT. Similarly, HIV treatment and related issues affect patients' response to IPT treatment; whereby patients would not be likely to adhere to IPT treatment if they are not willing or able to disclose their HIV status. Figure 2 illustrates how the five major themes interact.

### Integrating the Qualitative data with Quantitative data

As clearly evidenced in Table 1, included studies also collected quantitative data. When making informed decisions, local policy-makers need to examine a mix of quantitative and qualitative evidence. The high heterogeneity of the quantitative data, both in terms of methodological and statistical heterogeneity, does not allow the pooling together and/or meta-analysis of reported outcomes. In this respect, the included studies are found to be highly variable in terms of: their definition of the outcome measure (adherence rate); the demographic characteristics and other factors they report and analyse; and whether they report demographic characteristics by outcome (adherent versus non adherent groups). Still, while preserving the qualitative focus of our review, the team believes that it would be helpful to “triangulate” the thematic analysis with this quantitative data. Hence, the various factors in our model (Figure 2) are consequently revisited here to corroborate findings from the qualitative synthesis. Although the quantitative data are selective in the topics that they specifically address and, particularly, derive from a limited number of included studies, they do provide a mechanism for exploring the robustness of the synthesis [28].

### Personal characteristics

Personal factors related to people's characteristics such as age and sex are believed to have a major influence on their adherence behaviour. Gust and colleagues observed that the case non-adherent group was younger (t = 58.2, P,0.0001), and included a greater proportion of men (×2(1) = 5.7, P = 0.017), and persons with higher education (×2(2) = 3.6, P = 0.170) [20]. Mosimaneotsile et al [22] and Ngamvithayapong et al [24] also found that women were more likely to be adherent than men. Substance use was also associated with poor adherence with Gust et al reporting that “the case non-adherent group … had a greater proportion of…persons who drank alcohol (×2(1) = 4.4, P = 0.036) [20].

The quantitative components of the included studies typically focus on demographic factors. Our qualitative synthesis recognises the importance of the interaction of such demographic factors with individual personal beliefs in influencing trends in adherence of patients to treatment regimes [29], [30], [31].

### Individual personal beliefs

Munseri and colleagues substantiate the subtheme relating to fear of the side effects of INH, documenting that INH toxicity (n = 3, 13%; peripheral neuropathy (n = 1), hepatitis (n = 2]), INH intolerance, such as nausea (n = 4, 17%), were all reported by participants [23]. Mindachew and colleagues found, unsurprisingly, that those who developed IPT related adverse effects were 93% less likely to adhere to their prescribed doses [21]. Gust and colleagues also found that ‘the case non adherent group … had a greater proportion of persons who experienced any difficulty with the regimen (×2(1) = 21.9, P,0.0001)’ [20]. In this line, 22% of participants in the study by Szakacs and colleagues agreed with the statement, “INH is dangerous to your health” [26]. Munseri and colleagues report that “Non completers were more likely to cite… fear of INH side effects (n = 1, 14%)” [23].

An interesting tension emerges from the study by Munseri and colleagues [23], whereby fear of TB itself is reported as a major explanation for the decision to complete IPT. They observe that an understanding of the importance of IPT (n = 35, 32%) was an important factor: “completers were more likely to … think IPT was important (100% vs. 87%, 95%CI 0.063–0.197, *P*<0.001)” [23]. In this context, knowledge of the importance of IPT to successful maintenance of health may prove critical, as Szakacs and colleagues also report that “84% agreed that without INH, your chance of getting sick from TB is high” [26].

While individual personal beliefs are critical, our interpretation is congruent with Munro et al, who emphasise interactions between these ‘personal factors’ and factors emanating from the other broad components, namely, socio-economic and health service factors [27]. A similar interpretation by one of the included studies exemplifies this assertion: In explaining the ‘paradoxical’ observation that ‘those believing they have a high or above average chance of active TB without INH were more likely non-adherent’, Szakacs and colleagues draw upon this concept of interaction, stating that the influence of stigma could be one factor interfering with the translation of patient understanding into positive action [26].

### HIV treatment and related issues

While the included studies include comparatively little quantitative data on many aspects of HIV treatment, some examples attest to the added complexity due to patients being treated for HIV and with preventive therapy for TB at the same time. For example, Gust and colleagues describe how “the case non-adherent group…had greater proportion of …persons who initiated ART (×2(1) = 1.70, P = 0.192)” [20]. In contrast, Mosimaneotsile and colleagues report that “those receiving ART over the first 6 months were 1.41 fold more adherent” [22].

### Family and other social support related factors

Quantitative evidence from the included studies also attests to how family and other support mechanisms play a significant role in adherence. Munseri and colleagues quantify how completers were significantly more likely to have a family member or friend with TB “(65% vs. 12%, 95% confidence interval (CI) 0.185–0.875, *P*<0.03)” [23]. Furthermore, completers were “more likely to… have family approval for their decision to take IPT (97% vs. 50%, 95% CI 0.1411–0.6389, *P*<0.001)” [23]. The spouse may be particularly influential in instigating, or at least rationalizing, non-adherence: “Non completers cited…spouse's advice (n = 1, 14%)” [23].

Where family and social support is lacking, there is a strong perception of stigma associated with TB. Munseri and colleagues report how a large percentage of non-completers cited “stigma related to TB (n = 5, 58%)” as an important factor in their decision to stop IPT [23]. An alternative perspective is offered by Bakari and colleagues who explain that “…exposure or speculation about one's HIV status by a spouse in a married relationship can easily be linked to extramarital sexual affairs, a fact that may culminate in marital disharmony or even a divorce” [19]. The study from Ethiopia reported that those who had “good feeling/comfortable to take IPT drug in front of others were 6 times more likely to adhere …” [21].

### Socio-economic factors

Structural factors, revealed as important in the qualitative synthesis, also occurred as explanation for adherence in the quantitative data. Specifically, completers were more likely “to … describe the clinic as close to their residence (72% vs. 43%, 95%CI 0.01–0.672, *P*<0.04)” [23]. Correspondingly, “non completers cited travel distance to the clinic (n = 1, 14%)” [23].

### Health provider related factors

Finally, adherence is facilitated by interactions with a clinician, specifically, interactions that include counseling: “Completers were more likely to…find counseling helpful (91% vs. 63%, 95% CI 0.0574–0.5086, *P*<0.007)” [23]. Indeed, “patients who had received explanation about IPT were 8 times more likely to be adherent…”[21].

## Discussion

This systematic review assessed the available evidence regarding factors that facilitate or hinder adherence to TB preventive therapy amongst PLWHA. The review has accordingly drawn upon the findings of qualitative, quantitative and mixed method studies, while the focus lay in analysing the qualitative evidence with use of the quantitative data to add to the comprehensiveness of the review. We used the thematic analysis method [17], [32] which involves an analytical stage where the themes identified by the primary studies are interpreted in light of conceptual and theoretical understandings in the field. The analytical themes generated in this regard, the equivalence of third order generalisations in a meta-ethnography, go beyond mere collation of the findings of the included studies as they are ‘the result of interrogating a descriptive synthesis by placing it within an external theoretical framework’ [32]. The theoretical framework in our case is represented by the specific questions that we aimed to satisfy through the review. It is further constructed from existing conceptual understandings of adherence to treatment as a complex and dynamic phenomenon that is determined by the interaction of wide ranging factors [25], [27], [33].

The eight included studies specifically examine factors affecting adherence to isoniazid as an agent of preventive therapy for TB amongst PLWHA. In undertaking our analysis, we particularly adopted the theoretical framework or ‘model’ developed by Munro et al in their systematic review of factors that facilitate or deter patients' adherence to TB treatment [27]. Their systematic review [27] is a generic meta-ethnography that studied both curative and preventive treatments across general populations with TB. Only two studies [24], [25] are included in both the analyses by Munro et al [27] and in our review. Indeed five of the remaining reports, [20], [21], [22], [23], [26], fall outside the date range of Munro et al's wider systematic review (1966–February 2005) while the study of a specific police officer population was presumably excluded from their review for focusing on this specific population [19].

By employing meta-ethnography and undertaking third order interpretations of the primary themes identified in the studies they included in their review, Munro et al ‘developed a model to depict [an] understanding of the main influences on adherence’ [27]. The model accordingly includes four broad components, namely: ‘structural, personal, and health service factors influencing adherence, as well as social context’ [27]. We used these broad ‘components’ to inform our categorisation of the primary themes identified in the included primary studies whilst being sensitive to new categorisations that emerge from analysis and translation of the data across the included studies. Accordingly, we generated five major, interacting themes (Figure 2), which we believe best represent the data. These include: individual personal beliefs, HIV/AIDS treatment related factors, socio-economic factors, family and other support structures, and factors related to health providers. Our review hence largely confirms the findings of the systematic review by Munro et al [27], while highlighting and emphasising issues found to be especially pertinent in the specific context of adherence to a prophylactic therapy for TB patients living with HIV/AIDS.

Socio-economic factors identified in this review, which represent wider societal influences over which a patient has little or no control, such as, issues related to poverty, competing social commitments and service availability (distance to facilities and supply of drugs), are also widely recognised as critical factors determining patients' health behaviour [27]. Other studies examining adherence to HIV and TB medication in developing countries also identify medication related costs, especially financial costs for transport and food, and competition from other livelihood responsibilities [34].

HIV treatment and related issues represent a critical aspect of our analysis of factors; a theme that is especially pertinent to the topic at hand as it reflects the complications arising from the management/prevention of co-infection with two chronic diseases that have multifaceted societal ramifications. The themes that were pertinent under this broad theme are: denial of HIV status, HIV disclosure, stigma, and attitudes towards concurrent treatment of TB and HIV/AIDS. The double burden of stigma due to TB and HIV has been documented to exacerbate and compound the already existing stigma due to HIV in communities, whereby individuals living with both TB and HIV are found to be more likely to perceive stigma regarding their condition and to have very low disease-specific knowledge, particularly with regard to its severity [29], [30].

Munseri and colleagues explain the low adherence rate for women compared to men in their studies, stating, ‘in our setting [Tanzania], IPT is linked to HIV, and women in this study do not want their HIV status to be disclosed in fear of separation from their spouses…’ [23]. This further illustrates the complex interactions between the different major factors whereby gender values in specific socio-cultural settings influence how stigma attached to TB and HIV plays out to induce a differential ability of men and women to disclose their status.

Regarding the influence of the concurrent administration of IPT with ART, one of the included studies [22] reported a potentiating role of ART for IPT adherence. Another study [20] revealed patients' perceptions of pill burden as a factor for non-adherence. Other studies have also highlighted the potential re-enforcing role of ART treatment programmes as they bring in more organisation and resources in terms of counselling and support, whereas patients would still perceive taking too many pills as a hurdle [35], [36]. This is further complicated in the case of IPT, compared to active TB treatment, as people are generally more inclined to adhere to treatment when they are symptomatic than when asymptomatic [20], [25], [35], [36].

‘Family and other social support related factors’ are further identified as a major theme in this review, whereby psychosocial support from families and other social ties, the level of stigma faced by patients as well patients' concerns for their families have been identified by studies as determinants of adherence trends [23], [24], [25]. The medication adherence literature widely recognises this important factor, especially when involving highly stigmatising diseases such as HIV and TB [27], [34], [37].

The context of a ‘resource poor setting’ and under-developed health systems in developing countries renders the final theme critically important: ‘relationships with health providers’ [20], [24], [25], [26]. Patients' interactions with physicians and other health service staff, such as counsellors, and the availability of services and supply of drugs, figured prominently in our review [20], [21], [23], [24], [25], [26]. This major factor also features in reviews of adherence to HIV and TB therapy [27], [33], [34].

This review is naturally constrained by limitations in the “thickness” of detail [38], and consequently in the depth of analysis contained in the included primary studies. The included studies were conducted under different contexts and do not investigate all relevant issues in depth. For instance, interactions between the major themes are suggested, but not always explored, across the included studies. Also, the role of gender in mediating stigma in societies is identified but not explored in depth. The quantitative data reported across the studies is found to be highly disparate; thereby not allowing the pooling and/or meta-analysis of the results reported across the included studies. In addition, it is not always easy to discern the authors' interpretations from the primary data they were reporting. Thomas and Harden state, ‘one issue which is difficult to deal with when synthesising ‘qualitative’ studies is ‘what counts as data’ or ‘findings'?’ [32]. Also, even though the studies were all conducted in developing countries, the context of each study is different (well funded trials and cohort studies versus routine services, differences in interventions, follow-up and so on).

The implications of our review for practice are multi-fold as it synthesises the ‘disparate’ evidence on TB preventive therapy in PLWHA. It updates, and provides a more specific synthesis of, a previous, generic systematic review on TB treatment in people living with HIV/AIDS [27]. What is the value of a qualitative systematic review of a specific preventive agent (i.e. isoniazid) for TB in PLWHA when a generic model of adherence has already been developed for prevention and treatment of TB in the general population [27]? To a certain extent this debate is analogous to the wider debate of “lumping” and “splitting” that persists for systematic reviews as a whole [39]. Decision-makers need to engage with evidence at the level that is most appropriate to the decision that they are facing. Health policy makers need an overall model of the factors to be overcome for successful implementation of a prevent–and-treat policy. Such a generalisable model can be provided by a meta-ethnography which makes rich use of the data for interpretation and conceptual development. Local decision makers also deal with specific implementation strategies, around a specific drug such as isoniazid, in a specific population living with HIV/AIDS. A thematic synthesis can thus identify specific factors that need to be overcome and will typically present individual study level data that can contribute to a greater understanding of context. The decision-maker will therefore be interested in how findings in their population of interest diverge from findings in a general population facilitating identification of any factors that require special consideration.

Our review offers several implications for research and practice. Many of these are consonant, or indeed extend, findings from Rowe and colleagues [25]. Interventions that may improve adherence to latent TB and HIV/AIDS care in resource poor settings would include provisions that facilitate attendance at the clinics, particularly in rural areas and for those populations requiring frequent follow up. It is clearly important to ensure that the clinic environment is welcoming for HIV-positive patients by maintaining confidentiality and encouraging staff to be friendly and supportive. Previous studies conducted in Kenya and Zambia, confirm that the poor quality of physicians' interpersonal skills negatively affect adherence to treatment [40], [41]. One of the limitations of this review, due to the use of patients as the primary informants in the primary studies, is that the methodology cannot usually identify the absence of important components such as clinical leadership and clear policy guidelines [25]. A complementary perspective would be offered by interviewing clinical staff delivering the services. Similarly the needs for training, supervision and support are also latent, except where specific issues (e.g. poor communication skills) are perceived by the patient.

Future studies need to incorporate and test existing theoretical and conceptual understandings of adherence to TB preventive therapy in PLWHA. They also need to seek to generate a more in-depth understanding of how these complex factors interact with one another. Implementation research on adherence improvement strategies, guided by models derived from a sound conceptual understanding of adherence, will be critical for improving the persistently low levels of adherence to IPT amongst PLWHA.

## Conclusion

Adherence to IPT in PLWHA is influenced by the interactions of multiple factors. Accordingly, no one single strategy, such as, patient education or financial support for patients, will help improve adherence rates. A multi-pronged strategy that incorporates current understanding of the complexity of the interactive factors at play and that seeks to engage patients through a holistic, participatory and contextually tailored support is critical.

Our systematic review of the available qualitative evidence confirms, from multiple studies across diverse contexts, that adherence to preventive therapy for TB is:

“underpinned by complex interactions between the health service and the social, economic, and cultural environment in which patients and their communities are immersed” [25]

Only by understanding this complex interplay of factors more clearly will healthcare decision-makers be able to achieve the level of adherence required in order to combat the threat posed by TB in patients living with HIV and AIDS.

## Supporting Information

Checklist S1 PRISMA Checklist.
(DOC)

Table S1 Ovid Medline Search Strategy.
(DOCX)
