# Supplementary material for: Factors Associated with Adherence to Treatment with Isoniazid for the Prevention of Tuberculosis amongst People Living with HIV/AIDS: A Systematic Review of Qualitative Data
Source: PLoS One. 2014 Feb 3;9(2):e87166. doi: 10.1371/journal.pone.0087166 (PMC3911939; doi:10.1371/journal.pone.0087166)
Supplement: Table S1 — Ovid Medline Search Strategy. (DOCX) [file pone.0087166.s002.docx]

|  | Ovid MEDLINE(R) In-process & Other Non Indexed Citations and Ovid MEDLINE(R) <1950 to August 2011>  [*Update following translation across platforms run on PubMed <1966 to December 2011>*] |
| --- | --- |
| **No** | **Search strategy** |
| 1 | qualitative studies or Qualitative studie$).mp. |
| 2 | qualitative reviews.mp. |
| 3 | metasynthesi$.mp. |
| 4 | meta-synthesis.mp. |
| 5 | qualitative OR findings OR interview$ |
| 6 | 1 or 2 or 3 or 4 or 5 |
| 7 | HIV infection.mp. |
| 8 | AIDS. mp |
| 9 | AIDS treatment .mp |
| 10 | Antiretroviral therapy.mp |
| 11 | ART.mp |
| 12 | HAART. Mp |
| 13 | 7 or 8 or 9 or 10 or 11 or 12 |
| 14 | Tuberculosis or TB).mp. |
| 15 | Pulmonary tuberculosis.mp. |
| 16 | TB treatment.mp |
| 17 | DOTS.mp. |
| 18 | Antitubercular agents.mp. |
| 19 | Isoniazid.mp |
| 20 | Isoniazid preventive Treatment.mp. |
| 21 | Isoniazid preventive Therapy.mp. |
| 22 | Isoniazid$ |
| 23 | IPT.mp |
| 24 | 14 or 15 or 16 or 17 or 18 or 19 or 20 or 21 or 22 or 23 |
| 25 | 6 and 13 and 24 |
| 26 | Adherence.mp |
| 27 | (adheren$ OR complian$) |
| 28 | 26 or 27 |
| 29 | 25 and 28 |
